# Ophthalmic Complications and Managements of Sulfur Mustard Exposure: A Narrative Review

**DOI:** 10.34172/aim.2022.100

**Published:** 2022-09-01

**Authors:** Seyed Hosein Ghavami Shahri, Mahdi Balali-Mood, Hamid Reza Heidarzadeh, Mojtaba Abrishami

**Affiliations:** ^1^Eye Research Center, Mashhad University of Medical Sciences, Birjand, Iran; ^2^Medical Toxicology and Drug Abuse Research Center, Birjand University of Medical Sciences, Birjand, Iran; ^3^Ocular Oncology Service, Department of Ophthalmology and Visual Sciences, University of Toronto, Toronto, Canada

**Keywords:** Cornea, Mustard Gas Keratopathy, Retina, Sulfur Mustard

## Abstract

Sulfur mustard (SM) is a lethal chemical agent that affects many organs, particularly the eyes, respiratory system and skin. Even asymptomatic patients with documented SM vapor exposure may develop organ disorder many years later. Patients with even minor signs in the acute stage may experience late complications that necessitate surgery. Early decontamination and conservative measures could help the patients and decrease the complications. Despite decades of research, there is still no effective treatment for either acute or long-term SM-induced ocular complications. Even after multiple medications and surgical procedures, the majority of patients continue to have symptoms. For dry eye, punctual occlusion, autologous eye drops, and aggressive lubrication are used; for persistent epithelial defects (PED), tarsorrhaphy, amniotic membrane transplant, and stem cell transplantation are used; for total limbal stem cell deficiency (LSCD), living-related conjunctivolimbal allograft and keratolimbal allograft are used; for corneal vascularization, steroids, non-steroidal anti-inflammatory drugs, and anti-vascular endothelial growth factor prescribed; and for corneal opacities, corneal transplantation is done. Platelet rich plasma and topical drops containing stem cell transplantation for LSCD, photodynamic therapy paired with subconjunctival or topical anti-vascular endothelial growth factors for corneal vascularization, topical curcumin and topical ciclosporin-A for dry eye, and orbital fat-derived stem cells for PED are all alternative treatments that can be suggested. Despite the experimental and clinical research on the complications of SM exposure over the past decades, there is still no effective treatment for eye complications. However, supportive medical and surgical management has been applied with relatively good outcome.

## Introduction

 Sulfur mustard (SM) is a highly reactive lipophilic alkylating compound that was used in the twentieth century as a potent debilitating and vesicant chemical warfare agent.^1-3^ Meyer invented pure SM in 1886, and the German army used it against English soldiers for the first time during World-War-I.^4^ SM was also applied in a large scale by the Iraqi army against the Iranian troops between 1983 and 1988, resulting in the death of 10 000 combatants and even innocent civilians. SM continues to pose a serious threat, in acts of terrorism or war, to civilians and military personnel.^5^ Despite decades of research, there is still no effective treatment for either acute or long-term SM-induced ocular complications.^6-8^

 SM affects many organs, including the eyes and skin, respiratory, hematological and immune systems.^9-11^ It may be also absorbed through the digestive system after consuming contaminated food. Large doses of SM can damage the rapidly-growing cells in the bone marrow, resulting in short- and long-term immune system impairment.^9,12^ The primary toxic effects of SM usually appear after a variable period of delay, from 2 to 24 hours, depending on the mode of exposure, exposure length, and SM concentration, as well as environmental variables such as temperature.^2,12^

 The eyes are highly vulnerable to local SM trauma, with a threshold of 12 mg-min/m^3^ compared to 200 mg-min/m^3^ for the skin, and even low doses result in incapacitation and visual impairment.^13^ Acute ocular complications are characterized by photophobia, eyelid erythema, blepharospasm and edema, chemosis, corneal epithelial erosions, anterior segment inflammation, and subconjunctival hemorrhage.^13-15^ However, some patients develop more severe corneal pathologies, such as chronic keratitis, reduced corneal sensation, recurrent/persistent corneal erosions, limbal vasculature injury, and neovascularization, which may lead to significant vision loss and even blindness.^3,16^ These pathologic disorders are called mustard gas keratopathy (MGK), which manifests itself either immediately after exposure in the form of recurrent smoldering inflammation (chronic form) or after a clinically latent duration of 0.5-40 years in the delayed-onset form.^15-20^

## Brief Toxicology

 SM is an oily, straw-colored substance that smells like onions, garlic, or mustard.^13^ Since this substance is an aerosol of tiny oily droplets, the name “mustard gas” is misleading.^15,21-24^

 The incapacitating properties of SM are far more important than its ability to kill at a 50% lethal dose (LD50).^23,25^ When swallowed, the LD50 is around 200 mg, 4–5 g when applied to bare skin for an extended time, and 1500 mg-min/m^3^ when inhaled.^3,12^

 The SM alkylates products with DNA and proteins, such as albumin and hemoglobin. Its urinary metabolites were demonstrated to be helpful targets for detecting exposure to SM in humans.^26-29^

## Ocular Involvement

 The eyes are the most vulnerable body organ affected by SM. The vulnerability is due to the moisture of the ocular surface, the severe lipophilicity of the gas, and the high turnover rate in the capillary electrochromatography, and metabolic activity.^3,22^ Between 75% and 90% of SM exposed individuals have ocular involvements. After 2-6 weeks of SM exposure, the acute eye symptoms usually improve without causing any more inflammation. Photophobia, on the other hand, may last a longer time. A continuous process may develop in a minority of patients, presenting as constant smoldering inflammation (chronic form) or late-onset lesions (delayed form) that appear a considerable number of years after a variable silent period.^3,16^

 It is still unknown whether SM triggers a continuous cascade of gradual ocular surface inflammation that progresses at various rates in different individuals. In long-term effects, severe acute lesions fade away; however, symptoms like dry eye, foreign body sensation, and photophobia persist, and sequelae like limbal ischemia, corneal epithelial erosions, and, in rare cases, peripheral corneal thinning and/or neovascularization can progress gradually.^16^ Patients in the delayed phase of the disease become asymptomatic and have their lesions cured within weeks of exposure, simply to see the symptoms reappear years later.^3,16^

## Acute Stage

 The severity and timing of an allergic reaction are influenced by the concentration, duration, and dose of SM exposure.^30,31^ Clinical symptoms one hour after exposure include progressive soreness, a bloodshot appearance, and grittiness before progressing to edema and acute conjunctivitis. After two to six hours of exposure, patients experience lacrimation, severe ocular pain, photophobia, reduced visual acuity, and blepharospasm.^23^

**Figure 1:**
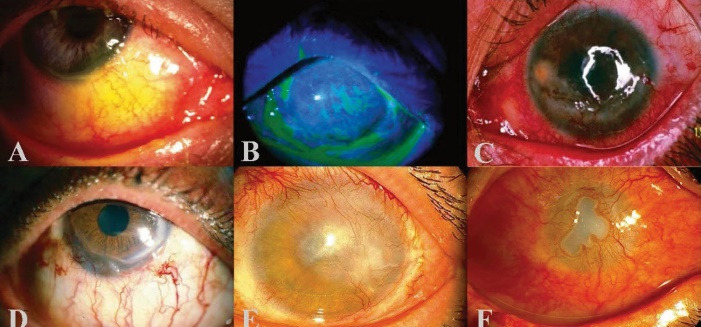
Sulfur Mustard (SM) Injury. A) Mild eyelid erythema, conjunctival congestion and B) diffuse punctate keratopathy seen at the acute phase of SM exposure. C) Limbal ischemia, corneal abrasion, peripheral neovascularization and lipid keratopathy following the acute phase of SM injury. D) Mild, E) moderate and F) severe stages of chromic SM injury

 Primary lesions are classified into three categories based on their severity: mild, moderate, and severe. Exposure to 12–70 mg-min/m^3^ causes mild erythema and swelling of the eyelids and conjunctival engorgement but not severe chemosis (Figure 1A). The cornea is typically spared, and recovery takes only a couple of days.^3,32^

 Exposure to SM at 100-200 mg-min/m^3^ causes moderate lesions. Lesions on the eyelids, conjunctiva, and cornea are similar to, but more serious than, the mild injury.^3,32,33^ Symptoms are a dry sensation in the eyes, severe ocular discomfort, severe blepharospasm, and photophobia.^3,15,26,34,35^ The corneal epithelium starts to slough and vesicates in the interpalpebral fissure, causing corneal abrasions, superficial punctate keratitis, superficial infiltrations, corneal ulcers, and even perforation (Figure 1B). The superior cornea is relatively unscathed, owing to the upper lid’s protective effect. Pain and blepharospasm usually fade after 48 hours, and the corneal epithelium heals fully within four to five days. It could take up to six weeks or longer for symptoms to disappear completely.^3,13,23,33,36^

 Severe lesions develop when exposure to SM is at a rate of higher than 200 mg-min/m^3^. Patients exposed frequently to such substances, develop more systemic toxicity. The deeper corneal layers and limbal vasculature are also involved, in addition to the moderate lesions. Ulcers may form on the eyelids. The temporal and nasal limbi lose their normal blood supply, resulting in white necrotic tissue. Necrosis and ischemia, especially in the interpalpebral fissure, chemosis and congestion, characterize severe conjunctival lesions.^3,37^ Conjunctival lesions are confined to the interpalpebral fissure, so adhesion between the eyelid and globe is impossible.^37^ Low-grade iridocyclitis with no cataract or synechia formation may occur, and intraocular pressure can briefly rise.^3,24,33^ When anterior uveitis develops, it can cause pupillary constriction, hemorrhages, necrosis, and iris vasodilation. An orange peel appearance is caused by a combination of stromal edema and corneal epithelial irregularity. Nevertheless, epithelial erosions and minor corneal ulcers can be evident. Pseudomonas aeruginosa super-infection may cause serious intraocular infection, leading to evisceration or immediate tectonic penetrating keratoplasty (PKP). The cornea sensation can be influenced in a variety of ways. When the uveitis subsides, and the corneal edema resolves, it usually takes 1-2 weeks for things to get better. The neovascularization process starts after a few weeks. The corneal stroma and subepithelial space are prone to bleeding from these tortuous blood vessels. They deteriorate quickly and leave white opacities in normally transparent corneas.^3,37^

## Chronic Phase

 Continuous delayed injuries occur in less than 1% of people exposed as late as 40 years following exposure. On the other hand, they usually result in lifelong loss of visual acuity and may also result in blindness. Chronic symptoms include foreign body sensation, injection, photophobia, and tearing. Meibomian gland dysfunction, lipid and amyloid accumulation, chronic blepharitis, limbal ischemia, dry eye, corneal neovascularization, limbal stem cell deficiency (LSCD), and corneal thinning, scarring, and irregularity are all distinguishing characteristics (Figure 1C).^3,23,36,37^ Descemetoceles and perforation stem from corneal irregularity, thinning, and neovascularization, diminished corneal sensation, compromised limbal vasculature, and frequent epithelial erosions.^3,23,36-39^ Corneal neovascularization can cause plasma lipids to exude into the stroma and amyloid formation.^37^

 The eyes may appear to be quiet despite the ongoing inflammation. However, this should not be mistaken for quiescence, as it could indicate ischemia due to vascular necrosis. Patients suffering from delayed-onset injury experience foreign body sensations, redness, severe photophobia, and tearing after a quiet duration of many years. The limbal region also has a marbled appearance in the early stages of ischemia, with porcelain-like regions of ischemia amid irregular-diameter blood vessels.^3,25,37,38^ Perilimbal ischemic areas are surrounded by tortuous, varicose, overflowing, and ampulliform vessels, covered by hemorrhages and blood islands. Lipoid depositions, as well as stromal inflammation and thinning, can occur in the adjacent cornea.^3,16,40,41^ Later, crystal and cholesterol deposits cover vascularized corneal scars, causing opacification to worsen, recurrent ulcerations, and occasionally corneal perforation.^26,42-46^ The lower and central sections of the cornea are commonly opacified, whereas the upper sections are frequently shielded by the eyelids. Even after corneal transplantation, these lesions can resurface. Corneal perforation and phthisis-bulbi may occur in a small number of patients.^26,47^

 Intrastromal corneal/conjunctival hemorrhages and blood islands, ischemic conjunctival areas, aberrant neovascularization, and lipoid and amyloid depositions are all possible signs of mustard gas-induced chronic vasculitis. Chronic conjunctival/corneal inflammation may cause chronic blepharitis with thickening of the lid margins and dysfunction of the meibomian glands.^30^

 Three levels of mild, moderate, and severe ocular surface involvements have been described for the chronic stage. The mild form is characterized by changes in conjunctival vessels (tortuosity, segmentation, and telangiectasia), as well as a clear adjacent corneal quadrant (Figure 1D). The moderate form is characterized by limbal ischemia/peripheral vessel invasion, and corneal opacity (Figure 1E). Severe involvement is defined as previous findings combined with severe corneal thinning/melting (Figure 1F). The clinical image of SM-induced ocular surface disorder has a wide range, making severity grading impossible.^45,48^

 In most severe cases, LSCD begins gradually in mild forms and progresses to total LSCD. Several underlying mechanisms appear to be involved in the late clinical manifestations of MGK. LSCD improves from partial/asymmetric to complete LSCD due to the immediate and progressive adverse effects of chronic limbal ischemia or mustard gas. Chronic ischemia can disrupt the stem cell niche, leading to stem cell attrition. Corneal nerve damage, which results in corneal sensation loss, contributes to the long-term negative effects of this condition.^45-49^ After exposure, nerve fiber degeneration with typical Wallerian degeneration was observed in an animal study, which lasted several weeks to several months.^50^

 Autoimmune responses to corneal antigens modified by SM (collagen-mustard complex) have been suggested. The corneal trophic/neurotrophic changes, such as descemetocele formation, perforation, and thinning, are caused by limbal ischemia in delayed MGK. The role of identified angiogenic factors in angiogenesis induced by SM is still unclear. Inflammation may contribute to propagation of the SM-delayed response. Anti-inflammatory therapy reduces the primary inflammatory response and the extent of neovascularization in animals.^49-56^

 All veterans of corneal injuries, such as alkaline/acidic burns, have a similar clinical course. The mystery of why some eyes are more susceptible to SM’s delayed response remains unsolved. Individual proximity to the ground, personal susceptibilities such as underlying disease, age, and immune system contribute to vulnerability. Because of children’s thinner skin and their proximity to the dirt, where mustard vapors collect, they are usually more seriously affected.^26,45,48,57,58^

 Reports of delayed responses to SM exposure may also be influenced by the form and length of follow-up. A perplexing characteristic of delayed MGK is the unpredictability of remissions and exacerbations in the clinical phase, with peripheral corneal infiltrates spreading to the center, mimicking Mooren’s ulcer.^45,58,59^ Corneal infiltrates are often caused by neovascularization, which resembles conjunctival vessels morphologically and is a weak prognostic factor. Intrastromal hemorrhages and perforation are possible side effects of infiltrations. Centripetal and deeper infiltrations, as well as degenerative modifications and crystalline deposits, are all signs of recurring episodes.^26,43,45,59^

 MGK is triggered by a lack of stem cells, which is one of the leading causes. Corneal manifestations are not characteristic of full or partial LSCD observed in severe chemical burns. The cornea is not entirely vascularized in SM-induced keratopathy; instead, corneal thinning with lipoid/amyloid depositions can be seen. The peripheral cornea is invaded in certain areas by leaking telangiectatic vessels. The corneal involvement is usually asymmetric between the two eyes, and its severity in each eye varies across quadrants. The interpalpebral exposed corneal layer is usually badly affected since it is close to conjunctival ischemic zones. It may be attributed to close interaction of the interpalpebral fissure at the time of exposure or more intense perilimbal ischemia in these regions. Perilimbal ischemia, in turn, could be caused by direct tissue exposure, resulting in vasculitis.^48^

 Finally, corneal manifestations tend to be a combination of chronic limbal ischemia and lack of stem cells. Exposure patterns could explain the differences in the clinical picture. Chemical burns expose LSCs to high concentrations of acid/alkali liquids, whereas SM targets the ocular surface to aerosolized gas. The pathophysiologic mechanisms and clinical pictures of SM-induced keratopathy have been studied in some animal studies, but they are beyond the scope of this review.^43,50,60^

## Retinopathy

 Shoeibi et al studied 40 highly intoxicated Iranian veterans’ retinal electrophysiological responses. They concluded that SM toxicity had delayed toxic effects in the retina but not in the retinal pigment epithelium layer. Since the eye is made up of neural tissue, SM is expected to have long-term effects on neural tissues.^61^

**Figure 2:**
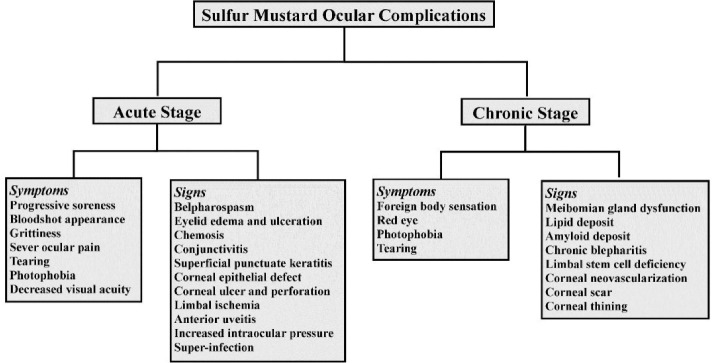
Summary of Symptoms and Signs of Ocular Effects in Acute and Chronic Stages of Sulfur Mustard Exposure

- The ocular symptoms and signs of SM exposure in the acute and chronic phases are summarized in a flow chart in Figure 2.

## Ocular Injury Management

###  Acute Phase

 Fluorescein staining initiated instantly after ocular washing can distinguish between conjunctival and corneal involvement. Preservative-free lubricants, topical corticosteroids, and antibiotics are among the medical treatments used at this stage. When there is corneal involvement, it is necessary to keep a close eye for the patient.^3,25^

 Mydriatics (to alleviate ocular discomfort caused by ciliary muscle spasm and avoid posterior synechiae), topical anti-glaucoma prescriptions (to control intraocular pressure), and antibiotic drops (to prevent bacterial super-infections) are also recommended therapies.^3,39,62^

 Since lubricants can accumulate SM particles stuck beneath the eyelids, they are controversial. Ocular bandage may increase the corneal temperature and hasten the toxic effects. Thus, it should be done only if seriously required. Topical corticosteroids can improve conjunctiva, cornea, and eyelid swelling, and anterior uveitis. However, their frequent use may make the cornea more susceptible to infection. To avoid sticking, petroleum jelly is applied, but SM can become concentrated in such an oily medium. Therefore, it should not be used immediately after SM exposure. Because the eye lesions cause fear and severe photophobia, dark glasses and patient assurance are essential.^3,13,25^

 Amniotic membrane transplantation (AMT) may reduce inflammation and scarring while promoting healing. When used in the acute phase of Stevens-Johnson syndrome and acute chemical burns, its soothing and anti-inflammatory properties have been demonstrated to avoid late sight-threatening cicatricial complications. As a result, it may be helpful in treating SM-related ocular surface disorders in their early stages.^63-73^

###  Chronic phase

####  Medical Treatment

 There has been no definitive treatment for ocular surface disorders caused by SM that are chronic and delayed.^3,16,25,46^ Currently, tear loss and ocular surface dysfunction are treated with blepharorrhaphy, artificial tears, tarsorrhaphy, therapeutic contact lenses, and temporary or permanent punctal occlusion. Since the ocular surface is weakened, particularly in the severe types of SM injury, artificial tears or gels that are preservative-free should be used. Punctal plugs are often advantageous. Electrocautery may be preferable for permanently occluding one or both puncta of each eye.^36,38,41^

 Topical steroids should be employed to combat both acute episodic corneal inflammatory infiltrates, and ocular surface inflammation. Topical steroids without preservatives are advantageous, especially when used for a short time. Although systemic steroids are rarely required, they may be used in conjunction with topical steroids in the event of severe corneal inflammation. Long-term steroid-induced complications, such as glaucoma and cataracts, should be taken into account. Because mustard gas keratitis causes loss of corneal integrity, the ocular surface is vulnerable to secondary microbial infection; thus, topical steroids need to be used with caution.^3,16,41,74^

 High DK silicone hydrogel contact lenses could assist with punctate epithelial erosions/keratitis and persistent epithelial defects (PEDs) in the case of partial LSCD. They are ineffective with total LSCD and can lead to microbial super infections. In all SM cases, blepharitis treatment is recommended. High DK hard/soft contact lenses and spectacles can be employed to improve visual acuity, but they should be used with caution. Conservative measures, such as topical/systemic steroids, are effective in recurrent and severe inflammatory infiltrates.^16,30,34,41,74^

## Artificial Tears and Lubricants

 Preservative-free artificial tears and lubricants have shown benefit for dry eye disease and corneal epithelial defects due to SM exposure. As a result, new artificial tear formulas have been designed, and their efficacy has been reported in treating dry eye disease of any etiology. One of the new formulas is hyaluronic acid with trehalose, which is safe and improves patient satisfaction.^47,67,75^

 Corneoconjunctival epithelial staining can be achieved by combining natural tear film components (hyaluronic acid, anionic glycosaminoglycan polysaccharide, carmellose sodium, and polymers). CHO-1 is a combination of carmellose sodium, osmoprotectants, and hyaluronic acid, to improve dry eye symptoms.^56^

 Recombinant human lubricant (Proteoglycan), a natural substance utilized as a lubricant, is an effective and safe agent. Artificial tears are one of the most commonly used treatments for SM patients with ocular symptoms.^3,54-56^

## Anti-inflammatory Drugs

 Various topical and systemic anti-inflammatory drugs are relatively effective in the treatment of SM-exposed victims. Corticosteroids, such as dexamethasone, and non-steroidal anti-inflammatory drugs (NSAIDs), such as diclofenac, are commonly prescribed to reduce SM-induced ocular inflammation.^3,52,53^

 Other anti-inflammatory drugs such as clobetasone,^51^ pranoprofen,^76^ bromfenac,^77^ and thymosin 4 eye drops,^78,79^ significantly contribute to the treatment of dry eye disease and corneal vascularization. As a result, these medications may be used in delayed ocular lesions.

 In the experimental model of SM exposure, concurrent utilization of corticosteroids and NSAIDs is also suggested. Ocular infection, glaucoma, and cataract have all been linked to long-term use of corticosteroids. Topical forms of thymosin 4, bromfenac, and pranoprofen have not been associated with ocular toxicity. Systemic steroids and topical steroids are used together in the event of severe corneal inflammation.^51,76-79^

 Cyclosporine-A refers to an immunomodulatory ophthalmic agent that has been used at varying doses (0.05%, 0.1%, 1%, and 2%) to improve tear production in patients with dry eye. The IL-2 signaling pathway is blocked by cyclosporine, which inhibits the immune response mediated by T-cells. Using a 0.05% concentration cyclosporine ophthalmic drop three times a day has shown benefit for SM-induced dry eye. In SM-exposed patients with dry eye disease, an increase in tear osmolarity is observed as a consistent finding. One of the leading causes of corneal nerve sensitivity and integrity loss in dry eye patients is tear film hyper-osmolality. This may lower the tear osmolality. Cyclosporine ophthalmic drop twice daily reduces lymphatic cell counts while increasing goblet cell counts in SM-exposed patients. In a mouse model of dry eye, Daull et al used a cationic cyclosporine-A emulsion 0.1%. They claimed that this novel formulation was more effective in treating dry eye than methylprednisolone 1%. Using a cationic cyclosporine-A emulsion in the treatment of corneal epithelium lesions was reported to be effective.^80-83^

 Tacrolimus is described as a valuable drug for treating keratoconjunctivitis, reducing ocular tissue inflammation, and improving tear film stability.^84,85^ Fingolimod is a systemic drug used to treat multiple sclerosis that is also used for treating dry eye disease. According to a mouse study, the safe doses of these two drugs are 0.005% and 0.1%. This novel formulation may be a long-term therapeutic alternative.^86^ In SM-exposed patients, dry eye treatment is vital for reducing ocular symptoms and enhancing quality of life.

## Surgical options

###  A. Tarsorrhaphy

 Medial or lateral tarsorrhaphy has been employed to stop the development of corneal thinning in SM patients with temporal or nasal progressive corneal thinning, whether or not they are taking PEDs. It may also help patients with persistent ocular surface inflammation and dry eye. It is strongly advised after any form of corneal or stem cell transplantations.^41,74^

###  B. Amniotic Membrane Transplant

 PEDs associated with partial LSCD could be treated with AMT. It is ineffective when LSCD is severe or complete. A combination of AMT and superficial keratectomy can be beneficial in seriously irritated eyes with severe photophobia caused by corneal lipid deposition. AMT can be also utilized as a graft or patch in conjunction with other stem cell transplants.^41,69,70,87-92^ It can be used in conjunction with medical treatment to relieve ocular surface inflammation, and scarring in cases of recurrent corneal inflammatory infiltrates. Corneas with severe thinning may benefit from multilayered AMT.^73,87,93,94^

###  C. Stem Cell Transplantation

 Patients with PEDs unresponsive to conservative treatments may be candidates for stem cell transplantation. Limbal regions close to the thinnest peripheral corneal locations of epithelial deficiencies are chosen as possible surgical sites. Simultaneous PKP or lamellar keratoplasty (LKP) is possible in some circumstances. LSCs can be harvested from living relatives, such as parents, siblings, or children (living-related conjunctival–limbal allograft, lr-CLAL), or cadaver eyes (keratolimbal allograft, KLAL). The matching of human leukocyte antigens is optional.^41,74^

 Different surgical procedures have been previously described.^41,95-100^ Tissue taken from one or both eyes of a close relative is fresher and has more similar genetic makeup than KLAL. However, a KLAL graft is more available and contains more stem cells. It is more subject to chronic stem cell attrition/rejection than an lr-CLAL graft, in addition to being less fresh. Because of the bilaterality, asymmetry, and partiality of LSCD, as well as differences in the magnitude of quadrant involvement, 360-degree full coverage of the limbal area by the graft is not necessary. In locations with the most severe corneal thinning and LSCD, sectoral KLAL/lr-CLAL appears to be sufficient. In the 40^th^ month, with an average follow-up time of 24.9 and 68.8 months, the rejection-free graft survival rates in the lr-CLAL community were reported to be 39.1% and 80.7%, respectively.^101^ Since patients may experience multiple systemic issues due to mustard gas exposure, only the bare minimum of immunosuppression is recommended. In some instances of severe ischemia, simultaneous ischemic conjunctiva resection and near-normal conjunctiva advancement can be helpful. It is unclear whether ischemia should be treated with conjunctival advancement or tenonplasty before stem cell transplantation.

###  D. Corneal Transplantation

 PKP or LKP can be adequate in cases where LSCD is mild and visual acuity is compromised because of central corneal opacification induced by lipoid/amyloid deposition.^37,74^ Perilimbal conjunctival ischemia is generally mild in these situations. Because of the recurrent and progressive aspect of the condition, it is recommended to spare the corneal endothelium and posterior stroma to avoid ocular surface derangements, and hence LKP is suggested. Severe corneal thinning, broad descemetoceles, and imminent or frank corneal perforation may necessitate the use of tectonic PKP. In cases of small descemetoceles, tectonic LKP may be used. Traditional LKP (deep anterior lamellar keratoplasty), Anwar, and Melles may be used. The rate of refuse-free grease survival was 39.0% in PKP patients and 90.3% in LKP patients, with the average follow-up time of 29.6 and 85.0 months.^50,101-103^ Because of corneal scarring, corneal thickness variability, and irregularity, the traditional LKP and Melles methods are frequently used. While corneal transplantation and LSC transplantation may be done simultaneously, it is recommended that these should be done at least three months apart. On account of the affected ocular surface, corneal transplantation in cases of SM-induced keratopathy is regarded to have a high risk.^74,87^ The cases with severe limbal ischemia and moderate LSCD with peripheral corneal thinning have a high graft loss owing to chronic opacity, rejection reactions, and corneal thinning.^41,74^

**Figure 3:**
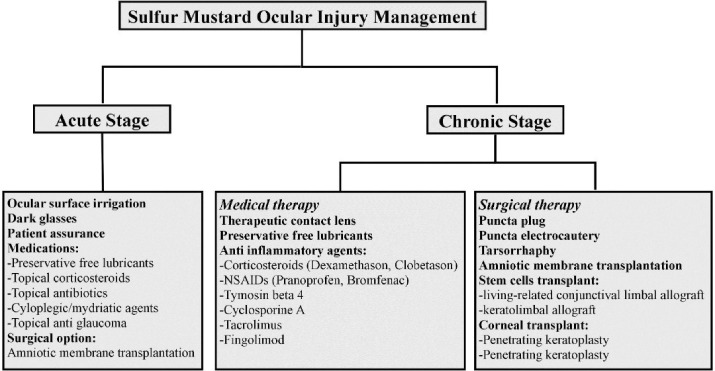
Summary of Management of Ocular Complications inAcute and Chronic Stages of Sulfur Mustard Exposure

 Management of ocular injury due to SM exposure in the acute and chronic phases is summarized in a flow chart in Figure 3.

## Novel and Potential Treatment Options for Mustard Gas Exposure

 While we now have a better understanding of how the cornea reacts to acute insults, lack of information about the etiology of chronic corneal disorders complicates the design of treatment strategies for treating SM injury.

 Topical corticosteroid and NSAID medications,^104^ colchicine,^105^ MMP inhibitors like doxycycline,^88^ and calcium-channel-blocker like diltiazem, have all been demonstrated to minimize ocular inflammation in animal studies. The use of nicotinamide, thiosulfate, flavonoids, and topically applied iodine has been linked to reducing dermal and systemic injury.^39,106-109^

 The acute ocular response to nitrogen mustard in rabbits was inhibited by colchicine.^105^ Treating rats with nicotinamide (a precursor to NAD + ) before exposure reduced the severity of SM-induced skin damage.^3,110^

 Thymosin 4 is a polypeptide that affects cell proliferation, differentiation, and migration, facilitate corneal healing, suppress inflammation, and modulate MMP activity when added ectopically.^36,58,79,111-113^ It has been suggested as an antidote in SM injuries.

 Highly reactive free radicals are known to be inhibited by metalocomplexes like zinc- or gallium-desferrioxamine. It is thought that either complex protects against acute phase damage by interfering with a crucial step in forming hydroxyl radicals. Topical iodine preparations and aminoguanidine, among other nitric oxide synthase inhibitors, have been shown to rescue or protect cells from SM-induced toxicity *in vitro*.^114^

 Ebelson, a peroxynitrite scavenger with no effect on nitric oxide synthase function, was discovered to be an important SM-induced toxicity inhibitor.^115^ A certain number of studies have demonstrated that glutathione or its N-acetyl-cysteine medicines can reduce SM- or SM analog-induced oxidative stress and toxicity.^49,59,115-119^

## Gene Therapy

 Current therapeutic techniques, such as immunotherapy, are believed to increase treatment efficacy by attacking malignant cells through various mechanisms. The current approach for treating various genetic diseases, especially cancers, is gene therapy, which involves injecting a manipulated gene into a defective cell.^120,121^ It includes two major strategies: (*a*) gene replacement, which involves the delivery of a normal gene to the cell and correcting functional protein, and (*b*) gene silencing, which involves using antisense RNA to make an overexpressed gene silent. By connecting to a specific mRNA location via base pairing in DNA molecules, this action performs transcription suppression in cells. Physical, chemical, and biological methods, such as bacteria, viruses, and exosomes, can all be used to deliver healthy and effective genes to target cells. These strategies can target tumor cells specifically while having minimal impact on healthy cells. Gene therapy, cancer vaccinations, and epigenetic agents do not entirely eliminate relapse of symptoms of certain cancers, including squamous cell carcinoma, melanoma, breast, kidney, and hematopoietic malignancies.^121-123^

 Managements of ocular complications inacute and chronic stages of SM exposure are summarized in Figure 3.

## Conclusion

 In conclusion, despite experimental and clinical research on the complications of SM exposure over the past decades, there is still no effective treatment for the eye complications. However, supportive medical and surgical management have been applied by different ophthalmology centers of Iran for the SM veterans with different outcomes that have been mostly palliatives.

## References

[1] Blodi FC (1971). Mustard gas keratopathy. Int Ophthalmol Clin.

[2] Graham JS, Schoneboom BA (2013). Historical perspective on effects and treatment of sulfur mustard injuries. Chem Biol Interact.

[3] Solberg Y, Alcalay M, Belkin M (1997). Ocular injury by mustard gas. Surv Ophthalmol.

[4] Fradkin EK (1928). Chemical warfare: its possibilities and probabilities. Int’l Conciliation.

[5] Smith KJ, Skelton H (2003). Chemical warfare agents: their past and continuing threat and evolving therapies Part I of II. Skinmed.

[6] Geraci MJ (2008). Mustard gas: imminent danger or eminent threat?. Ann Pharmacother.

[7] Saladi RN, Smith E, Persaud AN (2006). Mustard: a potential agent of chemical warfare and terrorism. Clin Exp Dermatol.

[8] Smith WJ, Dunn MA (1991). Medical defense against blistering chemical warfare agents. Arch Dermatol.

[9] Ghabili K, Agutter PS, Ghanei M, Ansarin K, Shoja MM (2010). Mustard gas toxicity: the acute and chronic pathological effects. J Appl Toxicol.

[10] Ghanei M, Amini Harandi A (2007). Long term consequences from exposure to sulfur mustard: a review. Inhal Toxicol.

[11] Kehe K, Szinicz L (2005). Medical aspects of sulphur mustard poisoning. Toxicology.

[12] Dacre JC, Goldman M (1996). Toxicology and pharmacology of the chemical warfare agent sulfur mustard. Pharmacol Rev.

[13] Dahl H, Gluud B, Vangsted P, Norn M (1985). Eye lesions induced by mustard gas. Acta Ophthalmol Suppl (1985).

[14] Mandel M, Gibson WS (1917). Clinical manifestations and treatment of gas poisoning. JAMA.

[15] Mann I (1944). A study of eighty-four cases of delayed mustard gas keratitis fitted with contact lenses. Br J Ophthalmol.

[16] Javadi MA, Yazdani S, Sajjadi H, Jadidi K, Karimian F, Einollahi B (2005). Chronic and delayed-onset mustard gas keratitis: report of 48 patients and review of literature. Ophthalmology.

[17] Ghasemi H, Ghazanfari T, Babaei M, Soroush MR, Yaraee R, Ghassemi-Broumand M (2008). Long-term ocular complications of sulfur mustard in the civilian victims of Sardasht, Iran. Cutan Ocul Toxicol.

[18] Ghasemi H, Ghazanfari T, Ghassemi-Broumand M, Javadi MA, Babaei M, Soroush MR (2009). Long-term ocular consequences of sulfur mustard in seriously eye-injured war veterans. Cutan Ocul Toxicol.

[19] Heckford F (1937). Delayed corneal ulceration following mustard gas burns. Proc R Soc Med.

[20] Moore RF, Heckford F (1929). Delayed corneal ulceration from mustard gas. Br Med J.

[21] Watson AP, Griffin GD (1992). Toxicity of vesicant agents scheduled for destruction by the Chemical Stockpile Disposal Program. Environ Health Perspect.

[22] Aasted A, Darre E, Wulf HC (1987). Mustard gas: clinical, toxicological, and mutagenic aspects based on modern experience. Ann Plast Surg.

[23] Balali-Mood M, Hefazi M (2005). The pharmacology, toxicology, and medical treatment of sulphur mustard poisoning. Fundam Clin Pharmacol.

[24] Safarinejad MR, Moosavi SA, Montazeri B (2001). Ocular injuries caused by mustard gas: diagnosis, treatment, and medical defense. Mil Med.

[25] Balali-Mood M, Hefazi M, Mahmoudi M, Jalali E, Attaran D, Maleki M (2005). Long-term complications of sulphur mustard poisoning in severely intoxicated Iranian veterans. Fundam Clin Pharmacol.

[26] Black RM, Brewster K, Clarke RJ, Hambrook JL, Harrison JM, Howells DJ (1992). Biological fate of sulphur mustard, 1,1’-thiobis(2-chloroethane): isolation and identification of urinary metabolites following intraperitoneal administration to rat. Xenobiotica.

[27] Black RM, Clarke RJ, Harrison JM, Read RW (1997). Biological fate of sulphur mustard: identification of valine and histidine adducts in haemoglobin from casualties of sulphur mustard poisoning. Xenobiotica.

[28] Black RM, Read RW (1988). Detection of trace levels of thiodiglycol in blood, plasma and urine using gas chromatography-electron-capture negative-ion chemical ionisation mass spectrometry. J Chromatogr.

[29] Wils ER, Hulst AG, van Laar J (1988). Analysis of thiodiglycol in urine of victims of an alleged attack with mustard gas, part II. J Anal Toxicol.

[30] Greenfield RA, Brown BR, Hutchins JB, Iandolo JJ, Jackson R, Slater LN (2002). Microbiological, biological, and chemical weapons of warfare and terrorism. Am J Med Sci.

[31] Smith KJ, Hurst CG, Moeller RB, Skelton HG, Sidell FR (1995). Sulfur mustard: its continuing threat as a chemical warfare agent, the cutaneous lesions induced, progress in understanding its mechanism of action, its long-term health effects, and new developments for protection and therapy. J Am Acad Dermatol.

[32] Balali-Mood M, Hefazi M (2006). Comparison of early and late toxic effects of sulfur mustard in Iranian veterans. Basic Clin Pharmacol Toxicol.

[33] Geeraets WJ, Abedi S, Blanke RV (1977). Acute corneal injury by mustard gas. South Med J.

[34] Mann I, Pullinger BD (1942). A study of mustard gas lesions of the eyes of rabbits and men. Proc R Soc Med.

[35] Kadar T, Turetz J, Fishbine E, Sahar R, Chapman S, Amir A (2001). Characterization of acute and delayed ocular lesions induced by sulfur mustard in rabbits. Curr Eye Res.

[36] Milhorn D, Hamilton T, Nelson M, McNutt P (2010). Progression of ocular sulfur mustard injury: development of a model system. Ann N Y Acad Sci.

[37] Javadi MA, Yazdani S, Sajjadi H, Jadidi K, Karimian F, Einollahi B (2005). Chronic and delayed-onset mustard gas keratitis: report of 48 patients and review of literature. Ophthalmology.

[38] Pleyer U, Sherif Z, Baatz H, Hartmann C (1999). Delayed mustard gas keratopathy: clinical findings and confocal microscopy. Am J Ophthalmol.

[39] Banin E, Morad Y, Berenshtein E, Obolensky A, Yahalom C, Goldich J (2003). Injury induced by chemical warfare agents: characterization and treatment of ocular tissues exposed to nitrogen mustard. Invest Ophthalmol Vis Sci.

[40] English F, Bennett Y (1990). The challenge of mustard-gas keratopathy. Med J Aust.

[41] Javadi MA, Baradaran-Rafii A (2009). Living-related conjunctival-limbal allograft for chronic or delayed-onset mustard gas keratopathy. Cornea.

[42] Eklöw L, Moldéus P, Orrenius S (1984). Oxidation of glutathione during hydroperoxide metabolism A study using isolated hepatocytes and the glutathione reductase inhibitor 1,3-bis(2-chloroethyl)-1-nitrosourea. Eur J Biochem.

[43] Fidder A, Moes GW, Scheffer AG, van der Schans GP, Baan RA, de Jong LP (1994). Synthesis, characterization, and quantitation of the major adducts formed between sulfur mustard and DNA of calf thymus and human blood. Chem Res Toxicol.

[44] Ghasemi H, Ghazanfari T, Yaraee R, Ghassemi-Broumand M, Soroush MR, Pourfarzam S (2009). Evaluation of relationship between the serum levels of inflammatory mediators and ocular injuries induced by sulfur mustard: Sardasht-Iran Cohort Study. Int Immunopharmacol.

[45] Noort D, Benschop HP, Black RM (2002). Biomonitoring of exposure to chemical warfare agents: a review. Toxicol Appl Pharmacol.

[46] Panahi Y, Rajaee SM, Sahebkar A (2017). Ocular effects of sulfur mustard and therapeutic approaches. J Cell Biochem.

[47] Pinto-Bonilla JC, Del Olmo-Jimeno A, Llovet-Osuna F, Hernández-Galilea E (2015). A randomized crossover study comparing trehalose/hyaluronate eyedrops and standard treatment: patient satisfaction in the treatment of dry eye syndrome. Ther Clin Risk Manag.

[48] Lim P, Fuchsluger TA, Jurkunas UV (2009). Limbal stem cell deficiency and corneal neovascularization. Semin Ophthalmol.

[49] Gould NS, White CW, Day BJ (2009). A role for mitochondrial oxidative stress in sulfur mustard analog 2-chloroethyl ethyl sulfide-induced lung cell injury and antioxidant protection. J Pharmacol Exp Ther.

[50] Jafarinasab MR, Zarei-Ghanavati S, Kanavi MR, Karimian F, Soroush MR, Javadi MA (2010). Confocal microscopy in chronic and delayed mustard gas keratopathy. Cornea.

[51] Aragona P, Spinella R, Rania L, Postorino E, Sommario MS, Roszkowska AM (2013). Safety and efficacy of 0.1% clobetasone butyrate eyedrops in the treatment of dry eye in Sjögren syndrome. Eur J Ophthalmol.

[52] Foulks GN, Forstot SL, Donshik PC, Forstot JZ, Goldstein MH, Lemp MA (2015). Clinical guidelines for management of dry eye associated with Sjögren disease. Ocul Surf.

[53] Jap A, Chee SP (2008). Immunosuppressive therapy for ocular diseases. Curr Opin Ophthalmol.

[54] Lambiase A, Sullivan BD, Schmidt TA, Sullivan DA, Jay GD, Truitt ER 3rd (2017). A two-week, randomized, double-masked study to evaluate safety and efficacy of lubricin (150 μg/mL) eye drops versus sodium hyaluronate (HA) 0.18% eye drops (Vismed®) in patients with moderate dry eye disease. Ocul Surf.

[55] Samsom ML, Morrison S, Masala N, Sullivan BD, Sullivan DA, Sheardown H (2014). Characterization of full-length recombinant human proteoglycan 4 as an ocular surface boundary lubricant. Exp Eye Res.

[56] Simmons PA, Carlisle-Wilcox C, Chen R, Liu H, Vehige JG (2015). Efficacy, safety, and acceptability of a lipid-based artificial tear formulation: a randomized, controlled, multicenter clinical trial. Clin Ther.

[57] McGahan MC, Bito LZ (1982). The pathophysiology of the ocular microenvironment I Preliminary report on the possible involvement of copper in ocular inflammation. Curr Eye Res.

[58] Sosne G, Christopherson PL, Barrett RP, Fridman R (2005). Thymosin-beta4 modulates corneal matrix metalloproteinase levels and polymorphonuclear cell infiltration after alkali injury. Invest Ophthalmol Vis Sci.

[59] Baradaran-Rafii A, Eslani M, Tseng SC (2011). Sulfur mustard-induced ocular surface disorders. Ocul Surf.

[60] Pfister RR, Haddox JL, Yuille-Barr D (1991). The combined effect of citrate/ascorbate treatment in alkali-injured rabbit eyes. Cornea.

[61] Shoeibi N, Mousavi MN, Balali-Mood M, Moshiri M, Darchini-Maragheh E, Mousavi SR (2017). Long-term complications of sulfur mustard poisoning: retinal electrophysiological assessment in 40 severely intoxicated Iranian veterans. Int J Retina Vitreous.

[62] Balali-Mood M, Mousavi S, Balali-Mood B (2008). Chronic health effects of sulphur mustard exposure with special reference to Iranian veterans. Emerg Health Threats J.

[63] Tejwani S, Kolari RS, Sangwan VS, Rao GN (2007). Role of amniotic membrane graft for ocular chemical and thermal injuries. Cornea.

[64] Arora R, Mehta D, Jain V (2005). Amniotic membrane transplantation in acute chemical burns. Eye (Lond).

[65] Bouchard CS, John T (2004). Amniotic membrane transplantation in the management of severe ocular surface disease: indications and outcomes. Ocul Surf.

[66] Dua HS, Gomes JA, King AJ, Maharajan VS (2004). The amniotic membrane in ophthalmology. Surv Ophthalmol.

[67] Fernandez KB, Epstein SP, Raynor GS, Sheyman AT, Massingale ML, Dentone PG (2015). Modulation of HLA-DR in dry eye patients following 30 days of treatment with a lubricant eyedrop solution. Clin Ophthalmol.

[68] Grueterich M, Espana EM, Tseng SC (2003). Ex vivo expansion of limbal epithelial stem cells: amniotic membrane serving as a stem cell niche. Surv Ophthalmol.

[69] Kheirkhah A, Casas V, Raju VK, Tseng SC (2008). Sutureless amniotic membrane transplantation for partial limbal stem cell deficiency. Am J Ophthalmol.

[70] Meller D, Tseng SC (1998). [Reconstruction of the conjunctival and corneal surface Transplantation of amnionic membrane]. Ophthalmologe.

[71] Prabhasawat P, Tesavibul N, Prakairungthong N, Booranapong W (2007). Efficacy of amniotic membrane patching for acute chemical and thermal ocular burns. J Med Assoc Thai.

[72] Sippel KC, Ma JJ, Foster CS (2001). Amniotic membrane surgery. Curr Opin Ophthalmol.

[73] Tseng SC (2001). Amniotic membrane transplantation for ocular surface reconstruction. Biosci Rep.

[74] Javadi MA, Yazdani S, Rezaei Kanavi M, Mohammadpour M, Baradaran-Rafiee A, Jafarinasab MR (2007). Long-term outcomes of penetrating keratoplasty in chronic and delayed mustard gas keratitis. Cornea.

[75] Chiambaretta F, Doan S, Labetoulle M, Rocher N, Fekih LE, Messaoud R (2017). A randomized, controlled study of the efficacy and safety of a new eyedrop formulation for moderate to severe dry eye syndrome. Eur J Ophthalmol.

[76] Liu X, Wang S, Kao AA, Long Q (2012). The effect of topical pranoprofen 0.1% on the clinical evaluation and conjunctival HLA-DR expression in dry eyes. Cornea.

[77] Yanai K, Huang J, Kadonosono K, Uchio E (2013). Corneal sensitivity after topical bromfenac sodium eye-drop instillation. Clin Ophthalmol.

[78] Sosne G, Qiu P, Kurpakus-Wheater M (2007). Thymosin beta-4 and the eye: I can see clearly now the pain is gone. Ann N Y Acad Sci.

[79] Yarmola EG, Klimenko ES, Fujita G, Bubb MR (2007). Thymosin beta4: actin regulation and more. Ann N Y Acad Sci.

[80] Daull P, Feraille L, Barabino S, Cimbolini N, Antonelli S, Mauro V (2016). Efficacy of a new topical cationic emulsion of cyclosporine A on dry eye clinical signs in an experimental mouse model of dry eye. Exp Eye Res.

[81] Hirata H, Mizerska K, Marfurt CF, Rosenblatt MI (2015). Hyperosmolar tears induce functional and structural alterations of corneal nerves: electrophysiological and anatomical evidence toward neurotoxicity. Invest Ophthalmol Vis Sci.

[82] Jadidi K, Panahi Y, Ebrahimi A, Mafi M, Nejat F, Sahebkar A (2014). Topical cyclosporine a for treatment of dry eye due to chronic mustard gas injury. J Ophthalmic Vis Res.

[83] Andrus L, Lafferty KJ (1981). Inhibition of T-cell activity by cyclosporin A. Scand J Immunol.

[84] Chatterjee S, Agrawal D (2016). Tacrolimus in corticosteroid-refractory vernal keratoconjunctivitis. Cornea.

[85] Moscovici BK, Holzchuh R, Sakassegawa-Naves FE, Hoshino-Ruiz DR, Albers MB, Santo RM (2015). Treatment of Sjögren’s syndrome dry eye using 0.03% tacrolimus eye drop: prospective double-blind randomized study. Cont Lens Anterior Eye.

[86] Xiao W, Sun L, Zhang N, Ye W (2017). Adverse effect profile of topical ocular administration of fingolimod for treatment of dry eye disease. Basic Clin Pharmacol Toxicol.

[87] Baradaran-Rafii A, Javadi MA, Rezaei Kanavi M, Eslani M, Jamali H, Karimian F (2010). Limbal stem cell deficiency in chronic and delayed-onset mustard gas keratopathy. Ophthalmology.

[88] Kadar T, Dachir S, Cohen L, Sahar R, Fishbine E, Cohen M (2009). Ocular injuries following sulfur mustard exposure--pathological mechanism and potential therapy. Toxicology.

[89] Lavker RM, Tseng SC, Sun TT (2004). Corneal epithelial stem cells at the limbus: looking at some old problems from a new angle. Exp Eye Res.

[90] Liang L, Sheha H, Li J, Tseng SC (2009). Limbal stem cell transplantation: new progresses and challenges. Eye (Lond).

[91] Liang L, Sheha H, Tseng SC (2009). Long-term outcomes of keratolimbal allograft for total limbal stem cell deficiency using combined immunosuppressive agents and correction of ocular surface deficits. Arch Ophthalmol.

[92] Sangwan VS, Tseng SC (2001). New perspectives in ocular surface disorders An integrated approach for diagnosis and management. Indian J Ophthalmol.

[93] Alió JL, Abad M, Scorsetti DH (2005). Preparation, indications and results of human amniotic membrane transplantation for ocular surface disorders. Expert Rev Med Devices.

[94] Hanada K, Shimazaki J, Shimmura S, Tsubota K (2001). Multilayered amniotic membrane transplantation for severe ulceration of the cornea and sclera. Am J Ophthalmol.

[95] Daya SM, Ilari FA (2001). Living related conjunctival limbal allograft for the treatment of stem cell deficiency. Ophthalmology.

[96] Dua HS, Azuara-Blanco A (1999). Allo-limbal transplantation in patients with limbal stem cell deficiency. Br J Ophthalmol.

[97] Espana EM, Di Pascuale M, Grueterich M, Solomon A, Tseng SC (2004). Keratolimbal allograft in corneal reconstruction. Eye (Lond).

[98] Kim JY, Djalilian AR, Schwartz GS, Holland EJ (2003). Ocular surface reconstruction: limbal stem cell transplantation. Ophthalmol Clin North Am.

[99] Solomon A, Ellies P, Anderson DF, Touhami A, Grueterich M, Espana EM (2002). Long-term outcome of keratolimbal allograft with or without penetrating keratoplasty for total limbal stem cell deficiency. Ophthalmology.

[100] Tsubota K, Goto E, Shimmura S, Shimazaki J (1999). Treatment of persistent corneal epithelial defect by autologous serum application. Ophthalmology.

[101] Javadi MA, Jafarinasab MR, Feizi S, Karimian F, Negahban K (2011). Management of mustard gas-induced limbal stem cell deficiency and keratitis. Ophthalmology.

[102] Alio JL, Shah S, Barraquer C, Bilgihan K, Anwar M, Melles GR (2002). New techniques in lamellar keratoplasty. Curr Opin Ophthalmol.

[103] Luengo-Gimeno F, Tan DT, Mehta JS (2011). Evolution of deep anterior lamellar keratoplasty (DALK). Ocul Surf.

[104] Amir A, Turetz J, Chapman S, Fishbeine E, Meshulam J, Sahar R (2000). Beneficial effects of topical anti-inflammatory drugs against sulfur mustard-induced ocular lesions in rabbits. J Appl Toxicol.

[105] Williams RN, Bhattacherjee P (1984). Inhibition of the acute ocular responses to nitrogen mustard by colchicine. Exp Eye Res.

[106] Elsayed NM, Omaye ST, Klain GJ, Korte DW Jr (1992). Free radical-mediated lung response to the monofunctional sulfur mustard butyl 2-chloroethyl sulfide after subcutaneous injection. Toxicology.

[107] Vijayaraghavan R, Sugendran K, Pant SC, Husain K, Malhotra RC (1991). Dermal intoxication of mice with bis(2-chloroethyl)sulphide and the protective effect of flavonoids. Toxicology.

[108] Vojvodić V, Milosavljević Z, Bosković B, Bojanić N (1985). The protective effect of different drugs in rats poisoned by sulfur and nitrogen mustards. Fundam Appl Toxicol.

[109] Wormser U, Sintov A, Brodsky B, Nyska A (2000). Topical iodine preparation as therapy against sulfur mustard-induced skin lesions. Toxicol Appl Pharmacol.

[110] Papirmeister B, Gross CL, Meier HL, Petrali JP, Johnson JB (1985). Molecular basis for mustard-induced vesication. Fundam Appl Toxicol.

[111] Sosne G, Chan CC, Thai K, Kennedy M, Szliter EA, Hazlett LD (2001). Thymosin beta 4 promotes corneal wound healing and modulates inflammatory mediators in vivo. Exp Eye Res.

[112] Sosne G, Qiu P, Christopherson PL, Wheater MK (2007). Thymosin beta 4 suppression of corneal NFkappaB: a potential anti-inflammatory pathway. Exp Eye Res.

[113] Sosne G, Szliter EA, Barrett R, Kernacki KA, Kleinman H, Hazlett LD (2002). Thymosin beta 4 promotes corneal wound healing and decreases inflammation in vivo following alkali injury. Exp Eye Res.

[114] Sawyer TW, Risk D (2000). Effects of selected arginine analogues on sulphur mustard toxicity in human and hairless guinea pig skin keratinocytes. Toxicol Appl Pharmacol.

[115] Laskin JD, Black AT, Jan YH, Sinko PJ, Heindel ND, Sunil V (2010). Oxidants and antioxidants in sulfur mustard-induced injury. Ann N Y Acad Sci.

[116] Ghanei M, Shohrati M, Jafari M, Ghaderi S, Alaeddini F, Aslani J (2008). N-acetylcysteine improves the clinical conditions of mustard gas-exposed patients with normal pulmonary function test. Basic Clin Pharmacol Toxicol.

[117] Kumar O, Sugendran K, Vijayaraghavan R (2001). Protective effect of various antioxidants on the toxicity of sulphur mustard administered to mice by inhalation or percutaneous routes. Chem Biol Interact.

[118] McClintock SD, Hoesel LM, Das SK, Till GO, Neff T, Kunkel RG (2006). Attenuation of half sulfur mustard gas-induced acute lung injury in rats. J Appl Toxicol.

[119] McClintock SD, Till GO, Smith MG, Ward PA (2002). Protection from half-mustard-gas-induced acute lung injury in the rat. J Appl Toxicol.

[120] Rafati-Rahimzadeh M, Rafati-Rahimzadeh M, Kazemi S, Moghadamnia AA (2019). Therapeutic options to treat mustard gas poisoning - Review. Caspian J Intern Med.

[121] Rahmani S, Abdollahi M (2017). Novel treatment opportunities for sulfur mustard-related cancers: genetic and epigenetic perspectives. Arch Toxicol.

[122] Ginn SL, Alexander IE, Edelstein ML, Abedi MR, Wixon J (2013). Gene therapy clinical trials worldwide to 2012–an update. J Gene Med.

[123] Janssen MJ, Arcolino FO, Schoor P, Kok RJ, Mastrobattista E (2016). Gene based therapies for kidney regeneration. Eur J Pharmacol.

